# Prognostic utility of late gadolinium enhancement on cardiac magnetic resonance in cardiac amyloidosis: a meta-analysis

**DOI:** 10.1186/1532-429X-18-S1-P115

**Published:** 2016-01-27

**Authors:** Sameer Raina, Shelly Lensing, Ramez Nairooz, Naga Venkata K Pothineni, Abdul Hakeem, Sabha Bhatti, Tarun Pandey

**Affiliations:** 1 grid.241054.60000000122929177Cardiology, University of Arkansas for Medical Sciences, Little Rock, AR USA; 2 grid.241054.60000000122929177Radiology, University of Arkansas for Medical Sciences, Little Rock, AR USA

## Background

Cardiac Amyloidosis (CA) is an important prognostic indicator in patients with systemic amyloidosis. Cardiac MRI has emerged as imaging modality of choice to evaluate patients with CA. Delayed hyper-enhancement cardiac magnetic resonance (DHE-CMR) provides incremental diagnostic and prognostic utility in suspected CA. We performed a meta-analysis to evaluate the prognostic role of LGE by CMR (LGE-CMR) imaging in patients with CA.

## Methods

Electronic databases MEDLINE, PubMed, Embase and Cochrane were systematically searched to identify studies evaluating the association between LGE-CMR and CA. The present study was designed to systematically review and assess the association between LGE and CA. The primary end-point was all-cause mortality. Pooling of odds ratios (OR) was performed using a random-effect model. Data were included from 7 studies with a total of 503 patients and a mean follow-up of 26 months.

## Results

Patients had a weighted average age of 62 years, LVEF of 57.6% and 62% were male. Cardiac Biopsy was positive for 21% patients while rest had evidence of positive extra cardiac biopsy, clinical and/or imaging features consistent for CA. LGE was present in 46% of patients. LGE positive patients had increased overall mortality compared to those without LGE (pooled OR 4.29, 95% CI 1.70 to 10.82, p = 0.002).

## Conclusions

LGE in CA patients is associated with increased risk of all-cause mortality; thus detection of LGE by CMR has prognostic implications which can provide risk stratification and further management in patients with CA.

**Figure 1:**
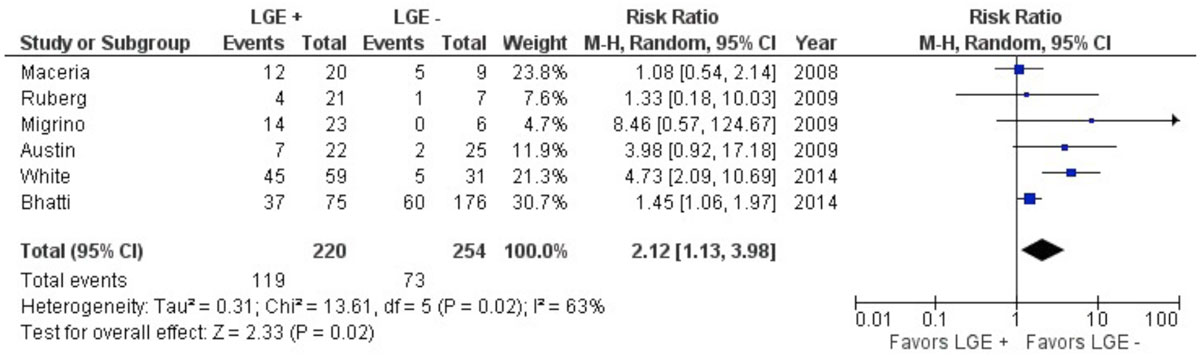
Figure 1

